# Unmasking the Basilar Culprit: A Case of Acute Posterior Circulation Stroke in a Diabetic Septuagenarian

**DOI:** 10.7759/cureus.79947

**Published:** 2025-03-03

**Authors:** Muhammad Rizwan Akram, FNU Veena, FNU Sabah Afroze, Matthew Peachey, Rene Elkin

**Affiliations:** 1 Internal Medicine, BronxCare Health System, New York, USA; 2 Neurology, BronxCare Health System, New York, USA

**Keywords:** basilar artery stenosis, cerebrovascular disease, diabetes mellitus, dual antiplatelet therapy, posterior circulation stroke

## Abstract

A 76-year-old man with poorly controlled diabetes mellitus, with an HbA1c of 10.9% (4.7%-6.4%), presents with acute right facial numbness, transient right eye foggy vision, and near syncope. Neuroimaging revealed substantial basilar artery stenosis with acute infarction in the region of the right posterior cerebral artery, impacting the medial temporal, occipital, thalamus, and midbrain. Although basilar artery stenosis is uncommon, its combination with poorly managed diabetes and posterior circulation involvement makes therapy difficult. Partial neurological recovery was observed with dual antiplatelet treatment and intensive risk factor management. Basilar artery stenosis has a poor prognosis; however, early detection, medical therapy, and comorbidity control can improve patient outcomes and reduce recurrence risk. The example further emphasizes the necessity of preventative treatment in high-risk patients and the link between diabetes control and posterior circulation stroke risk.

## Introduction

Basilar artery stenosis is an uncommon but potentially fatal cerebrovascular disease that affects around 1.43% of individuals suffering from acute ischemic stroke or transient ischemic attack [1]. The illness primarily impacts males in their sixties, with artery-to-artery embolism as the most prevalent stroke mechanism [1]. Posterior circulation strokes account for 20%-25% of all ischemic strokes, with severe basilar artery occlusive disease representing around 21.4% of posterior circulation incidents [1,2].

Basilar artery stenosis can manifest clinically in various ways, from minor symptoms to serious neurological impairments. Significant hazards are associated with the condition; research indicates that people with ≥50% basilar stenosis had a 46% 90-day probability of experiencing a transient ischemic attack or recurrent stroke [3]. Patients with ≥80% stenosis, mid-basilar placement, and poor collateral circulation face a higher risk [1].

We discuss the case of a 76-year-old patient with poorly controlled diabetes mellitus, who had abrupt neurological symptoms and was diagnosed with significant basilar artery stenosis, followed by a posterior circulation infarction. This case is notable because it highlights the complicated interplay between vascular risk factors and posterior circulation stroke, as well as the need for early detection and treatment of this potentially fatal illness [1,2].

The example also highlights the importance of complete vascular imaging in stroke evaluation and the need for aggressive risk factor management in high-risk groups to prevent repeated cerebrovascular episodes. Modern treatment strategies, such as dual antiplatelet therapy (DAPT) and high-dose statins, have shown promise in improving outcomes in these difficult situations [2,3].

## Case presentation

A 76-year-old man presented to the emergency room with a chief complaint of a sudden onset of right-sided facial numbness. The patient had a history of poorly controlled diabetes mellitus, with an HbA1c of 10.9% (4.7%-6.4%), hypertension, and long-term peripheral artery disease, resulting in the amputation of the right foot toes.

**Figure 1:**
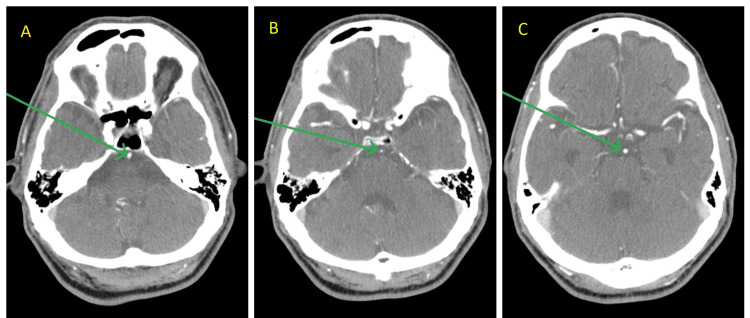
CT angiography head and neck Images (A)-(C) show short-segment severe stenosis of the basilar artery at three points (arrows), where maximum stenosis is apparent in Image (B). CT: computed tomography

**Figure 2:**
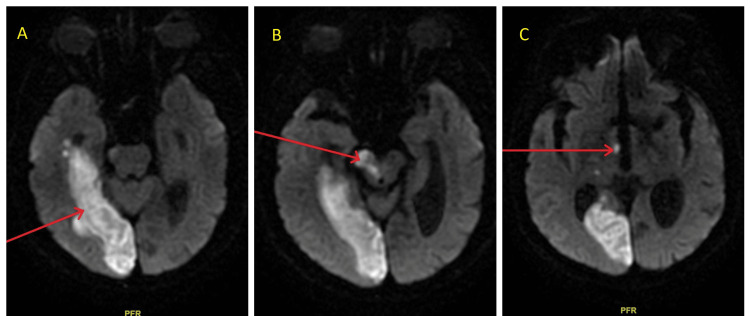
MRI brain without contrast Images (A)-(C) show acute or early subacute infarction (arrows) in the right posterior cerebral artery (PCA) territory, involving the medial temporal (A) and occipital lobes (A), as well as the midbrain (B) and the right thalamus (C). MRI: magnetic resonance imaging

The patient reported that his symptoms commenced at approximately 10 AM when he was in a pharmacy. He experienced a near-syncope episode, accompanied by hazy vision and a burning sensation in his right eye. The first vital signs showed a blood pressure of 154/50 mmHg, a heart rate of 71 beats per minute, a respiration rate of 18 breaths per minute, a temperature of 98.1°F (ca. 37°C), and an oxygen saturation of 100% in ambient air. Point-of-care glucose testing revealed hyperglycemia with a 431 mg/dL (normal, 70-120 mg/dL). An NIHSS (National Institutes of Health Stroke Scale) score of 2 was found on the neurological exam, along with signs of right-sided facial palsy affecting the lower face, tongue deflection to the right, and left-sided weakness, with motor strength of 2/5 in both the upper and lower limbs on the left side. The patient was alert and aware of his identity, surroundings, and temporal situation. A stroke code was initiated because the symptoms presented within 4.5 hours after onset. The preliminary non-contrast computed tomography (CT) scan of the head revealed no indications of an acute cerebral hemorrhage. However, stable encephalomalacia was observed in the left occipital lobe, and a lacunar infarct was observed in the left basal ganglia. CT perfusion imaging revealed a right frontal ischemic penumbra. CT angiography of the head and neck demonstrated a significant finding of short-segment severe stenosis of the basilar artery and a small V4 segment of the left vertebral artery (Figures 1A-1C). The follow-up magnetic resonance imaging (MRI) of the brain without contrast revealed an acute to early subacute infarction in the right posterior cerebral artery (PCA) territory, affecting the medial temporal and occipital lobes, and the right thalamus and midbrain (Figures 2A-2C).

The patient was hospitalized in the stroke unit and initiated on DAPT (aspirin and clopidogrel) in conjunction with high-dose statin therapy. Blood sugars were also well-managed while in the hospital. The neurosurgical evaluation determined that immediate surgical intervention was unnecessary.

The patient was recently hospitalized two months ago because of three observed seizures, accompanied by hyperglycemia and electrolyte abnormalities; nevertheless, the electroencephalogram (EEG) conducted at that time showed no epileptiform discharges. This recent history has introduced complexity to the contemporary presentation and management problems. The patient's health stabilized during hospitalization, with enhancement in right eye vision and preservation of stable neurological status. He required vigilant oversight and assertive alteration of risk factors, especially with his inadequately managed diabetes.

## Discussion

Basilar artery stenosis constitutes a rare yet significant cerebrovascular disorder, affecting approximately 1.43% of individuals experiencing acute ischemic stroke [1]. Case studies like this one show how difficult it may be to treat and predict outcomes when diabetes mellitus is poorly managed, and significant basilar artery stenosis is also present.

Research shows that people with diabetes are more likely to get posterior circulation ischemic strokes compared to those without diabetes [4], making the association between the two conditions all the more noteworthy. Since posterior circulation arteries differ from anterior circulation vessels in vasoreactivity, wall thickness, elastin content, and concentric intima thickening, these physiological variations may be responsible for the observed connection [4].

The clinical presentation of our patient is consistent with the established elevated risk of early recurrent stroke associated with vertebrobasilar stenosis. Research indicates that patients suffering from basilar or intracranial vertebral stenosis experience a 90-day recurrent stroke rate of 33%, which is markedly higher than the 16% rate noted for extracranial vertebral stenosis [5]. The existence of significant stenosis in the basilar artery, especially when coupled with inadequate glycemic control, significantly elevates the patient's risk for unfavorable outcomes.

Our treatment strategy conformed to contemporary evidence-based guidelines. Recent guidelines for high-risk scenarios endorsed the patient's DAPT with aspirin and clopidogrel [6]. The choice to forgo immediate stenting aligned with the results of the SAMMPRIS trial, which revealed that basilar artery stenting is associated with a significantly elevated risk of peri-procedural ischemic stroke (20.8% vs. 6.7% for other arteries) [7].

The extensive imaging methodology employed in this instance, encompassing CT angiography and MRI, exemplifies contemporary best practices. Recent studies have shown how important full vascular imaging is for the diagnosis and characterization of a stroke, especially when it comes to detecting basilar artery stenosis and checking for collateral circulation [8]. The acute infarction in the right PCA region was detected by MRI, which helped determine the patient's prognosis and course of therapy.

This case emphasizes the critical need for proactive risk factor management, particularly in managing diabetes. Studies demonstrate that diabetes increases the risk of stroke by 150% to 400%, especially in cases involving posterior circulation [9]. The patient's previous toe amputations highlight the systemic aspect of his vascular disease.

Recent data endorse the application of DAPT as more beneficial than single antiplatelet therapy when started early after a small stroke or high-risk transient ischemic attack, with a decreased 90-day risk of recurrent ischemic stroke [10]. For strokes not caused by blood clots in the heart, the European Stroke Organisation (ESO) guidelines strongly recommend short-term DAPT with aspirin and clopidogrel. This is especially important in cases with high-risk factors, such as intracranial stenosis [6].

In the past, medical treatment for basilar artery stenosis was thought to have poor outcomes. However, new research shows that modern therapeutic strategies can lower death rates to as low as 2.3%, with 75% of patients having mild or no deficits at follow-up [11]. The existence of inadequately managed diabetes and considerable stenosis may affect long-term prognosis.

This shows how important it is to continue researching the best ways to treat posterior circulation strokes, especially in people who have multiple risk factors. Some endovascular techniques have shown promising results in recent trials; however, medical care remains the most important method for treating basilar stenosis [12,13].

## Conclusions

This case demonstrates the complex interaction between poorly managed diabetes mellitus and basilar artery stenosis, which results in posterior circulation stroke. Basilar artery stenosis is rare but can have serious consequences; thus, it must be detected and treated promptly. We successfully treated our patient with DAPT (aspirin and clopidogrel) and aggressive risk factor modification. This case highlights the importance of employing a comprehensive treatment plan. It also shows that, although posterior circulation strokes with basilar stenosis remain challenging to manage, good results can be achieved with current therapies and close attention to other health issues that may be present.

This example shows that blood sugar control is crucial for preventing cerebrovascular accidents, particularly in the posterior circulation. Because of their numerous vascular risk factors, these patients require regular monitoring and strong preventive measures to avoid recurrent stroke and worsening cerebrovascular disease.
